# AI-driven bone mineral density prediction from chest x-rays and its association with obstructive sleep apnea

**DOI:** 10.1371/journal.pone.0330080

**Published:** 2025-09-05

**Authors:** Hung-Lung Tsai, Kuan-Hung Cheng, Po-Cheng Lin, Ying-Lin Hsu, Shih-Wei Chen

**Affiliations:** 1 Department of Information, Tungs’ Taichung MetroHarbor Hospital, Taichung, Taiwan; 2 Doctoral Program in Big Data Analytics for Industrial Applications, College of Science, National Chung Hsing University, Taichung, Taiwan; 3 Institute of Statistics, National Chung Hsing University, Taichung, Taiwan; 4 Department of Applied Mathematics, National Chung Hsing University, Taichung, Taiwan; 5 Department of Otolaryngology, Tungs’ Taichung MetroHarbor Hospital, Taichung, Taiwan; Xuzhou Central Hospital, The Xuzhou School of Clinical Medicine of Nanjing Medical University, CHINA

## Abstract

With an increasing aging population, the prevalence of chronic comorbidities is on the rise. The potential relationship between obstructive sleep apnea (OSA) and osteoporosis has garnered significant attention. Most studies examining the association between these two conditions have relied on dual-energy X-ray absorptiometry (DXA) to evaluate bone mineral density (BMD). Although DXA is considered the gold standard for BMD assessment, it does not reflect overall skeletal health. The limitations of conventional measurements are particularly pronounced in patients with multisystemic diseases, such as OSA. To address these limitations, we applied VeriOsteo™OP software to predict lumbar spine BMD and T-scores from chest X-ray (CXR) images. In addition, we examined the relationship between these bone health metrics and OSA. A total of 70,395 patients who underwent CXR examinations at Tungs’ Taichung MetroHarbor Hospital from 2017 to 2022 were included. Eligible samples were selected based on the presence of an OSA ICD-10-CM diagnosis code along with DXA results. By incorporating variables, such as gender, age, Body Mass Index (BMI), and T-score, we used multiple machine learning models, including logistic regression, random forest, and XGBoost, to analyze the risk for OSA. The results indicated that when the BMI range was controlled, the predictive contribution of the T-score became significant. For some models, the area under the curve (AUC) reached over 85% in both the training and validation datasets. This suggests a notable association between T-score and OSA, which is maintained when confounding variables such as BMI are controlled. This study highlights the potential of artificial intelligence (AI) technology using CXR imaging for osteoporosis screening. Combining CXR with machine learning models enables the assessment of OSA risk and offers a cost-effective, radiation-free screening tool with promising clinical applications.

## Introduction

The trend in rapid population aging is becoming increasingly evident worldwide, and Taiwan is no exception. Based on current projections, Taiwan is expected to officially become a super-aged society by 2025, with a significant increase in the proportion of elderly individuals. This demographic shift poses unprecedented challenges to the healthcare system, particularly for the management of age-related chronic conditions, such as obstructive sleep apnea (OSA) and osteoporosis [1].

In the elderly population, the prevalence of chronic disease comorbidities is escalating, with OSA and osteoporosis particularly concerning. The co-occurrence of these conditions is also increasing [2], which requires greater attention and targeted clinical interventions to address these dual health burdens effectively.

National surveys in Taiwan indicate a high prevalence of osteoporosis among men aged 50 years and older [3,4], which underscores the importance of addressing bone health management in male populations [5]. As age increases, the decrease in bone mineral density (BMD) significantly increases the risk of falls and fractures and poses a significant threat to the quality of life of elderly individuals [6–9].

OSA is also a major public health concern. According to data from Taiwan’s Ministry of Health and Welfare, approximately 5 million people in Taiwan are affected by sleep disorders and a significant portion suffer from OSA. The prevalence of OSA rises substantially with age. OSA is a common disorder with an increasing global prevalence, although the reported rates vary widely because of methodological differences. The prevalence of OSA defined at an apnea–hypopnea index (AHI) ≥5 was a mean of 22% (range, 9%–37%) in men and 17% (range, 4%–50%) in women in eleven published epidemiological studies published from 1993 to 2013 [10]. The prevalence increases with age, obesity, and male gender [11–13]. In certain elderly populations, the prevalence reaches 90% for men and 78% for women when AHI ≥ 5 [14]. Additional risk factors include elevated Body Mass Index (BMI), increased neck circumference, hypertension and smoking [15–19].

Although approximately 2% of women and 4% of men in Western populations are estimated to have OSA [20,21], there is a lack of systematic data on its prevalence in Asian populations [22]. Unfortunately, many OSA cases remain undiagnosed, leading to delayed treatment and potential adverse health outcomes. Studies indicate that as many as 90% of OSA cases in Canada remain undiagnosed [23]. Such diagnostic delays contribute to severe health consequences and increased healthcare costs [16,24].

The severity of OSA warrants attention because of its potential to contribute to intermittent hypoxemia, systemic inflammation, and metabolic dysregulation [25]. Of these factors, intermittent hypoxemia induced by OSA is a major contributor to the association between OSA and osteoporosis [13,26]. Intermittent hypoxia can elicit systemic inflammatory responses [27], activate osteoclasts, and inhibit osteoblast function, which leads to an imbalance in bone remodeling and subsequent bone loss [7,28,29].

In addition, OSA may influence bone health through other mechanisms, including hormonal imbalances (such as hypogonadism), oxidative stress, vitamin D deficiency, and dysregulation of glucocorticoids [30–32]. These mechanisms collectively contribute to the progression of osteoporosis [21,26,27,33].

The comorbidity of OSA and osteoporosis is projected to worsen with population aging. Approximately 936 million adults are affected by mild to moderate OSA worldwide, whereas 425 million adults have severe OSA requiring treatment [7,34]. These figures highlight the urgent need for more effective screening and early intervention strategies to address this challenge.

A study in Taiwan revealed that patients with OSA have a 2.74-fold higher risk of developing osteoporosis compared with non-OSA patients, which is a remarkable finding [35]; however, some meta-analyses have failed to identify a significant association between OSA and osteoporosis [36]. In addition, other studies suggest that in younger males (≤40 years), OSA-related moderate hypoxemia is positively correlated with higher BMD [37]. This suggests that the effect of OSA on bone health may vary depending on age and gender.

Studies predominantly rely on dual-energy X-ray absorptiometry (DXA) for measuring BMD. Although DXA is currently considered the gold standard for assessing osteoporosis [9,28,38,39], it has limitations for evaluating microstructural changes in bone and does not provide a comprehensive assessment of overall skeletal health, particularly in patients with multisystemic diseases, such as OSA. Moreover, DXA cannot distinguish between cortical and trabecular bone [40]. In addition, many studies utilize cross-sectional designs, making it difficult to identify the causal relationship between OSA and osteoporosis. Therefore, longitudinal studies are required to further verify the association between these two conditions.

The rapid advancement of artificial intelligence (AI) technology has introduced new applications for chest X-ray (CXR) in osteoporosis screening [9,41,42]. Studies have demonstrated that deep learning models can predict BMD and T-scores using CXR, with a high correlation with DXA results [43,44]. This AI-assisted screening approach not only reduces costs, but also offers effective osteoporosis risk assessment without increasing radiation exposure [44,45].

This study is the first to apply the VeriOsteo™OP software to predict lumbar spine BMD and T-scores from CXR images and to explore their association with OSA. This innovative technique overcomes the limitations of DXA, which measures only regional bone density, by offering a low-cost screening method that reduces the need for additional radiation exposure, making it particularly suitable for early screening in older populations [43,44]. Our research aims to provide a novel clinical tool to facilitate the combined screening of both OSA and osteoporosis at an early stage.

## Materials and methods

### Data resources

This was a cross-sectional, retrospective study that included patients who underwent CXR examination at Tungs’ Metro Harbor Hospital from January 2017 to December 2022. Initially, 70,395 CXR-PA images were collected, and the image dataset was subsequently transformed into a tabular dataset. The tabular data consisted of X-ray PA view images taken from 2017 to 2022, which were analyzed using VeriOsteo™OP software to obtain BMD and T-scores. The data were sourced from the electronic medical record system of Tungs’ Metro Harbor Hospital and spanned a six-year data collection period to ensure that the data was diverse and representative. The data used in this study were accessed on February 1, 2024, from the database of Tungs’ Metro Harbor Hospital. This study was conducted in accordance with the principles of the Declaration of Helsinki and followed current scientific guidelines. As the data were fully anonymized to protect patient confidentiality, the requirement for informed consent was waived.

### Ethical approval

The study was approved by the ethical committee of Tungs’ Taichung MetroHarbor Hospital in Taichung, Taiwan (protocol number 112069). Written informed consent was received from all patients.

### Inclusion and exclusion criteria

**Fig 1:**
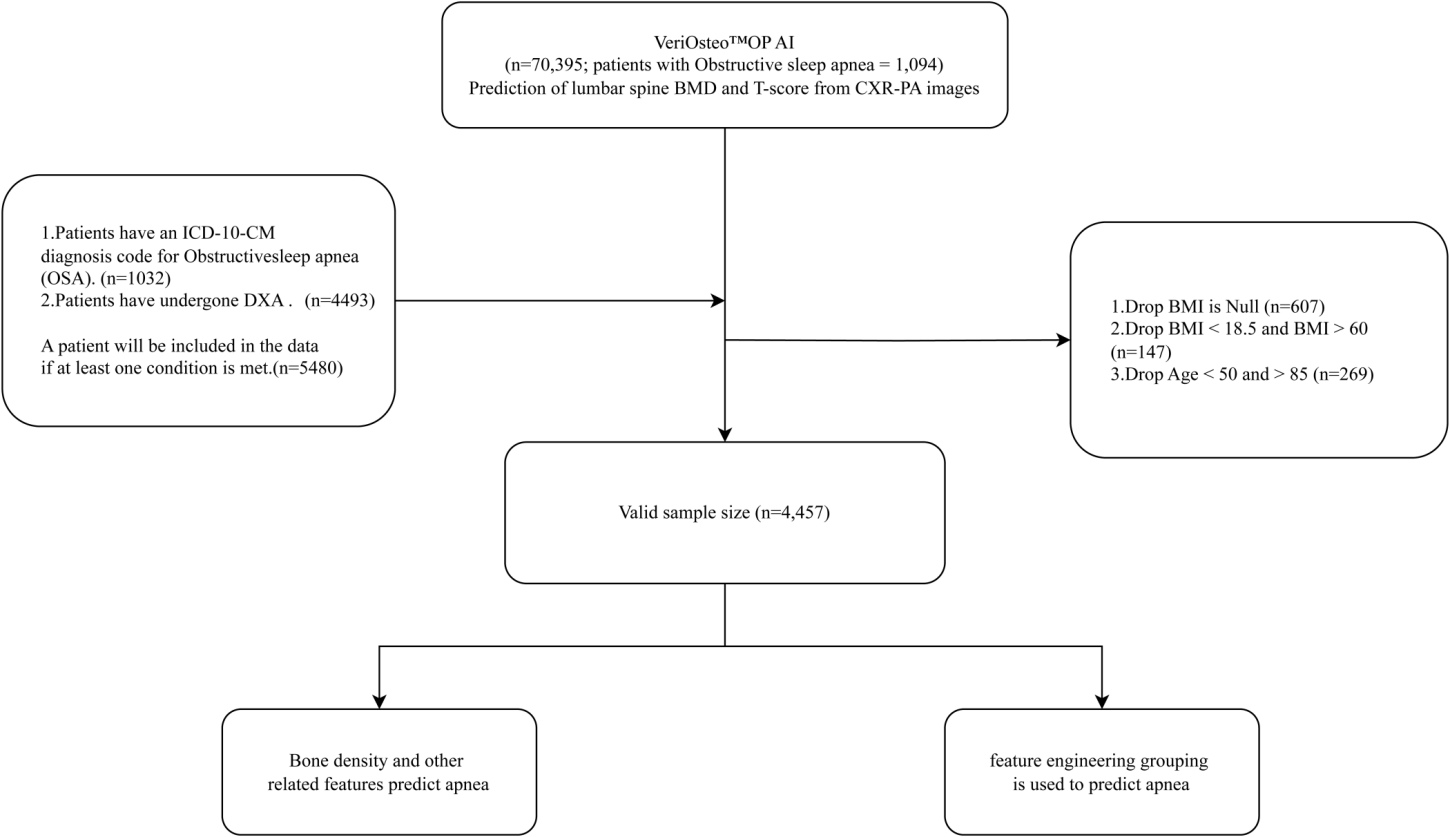
Research flow chart.

To ensure data quality and the reliability of the results, multiple exclusion criteria were established. First, patients had to meet at least one of the following conditions: (1) having a diagnosis of OSA confirmed by the ICD-10-CM code G47.33, or (2) having undergone a DXA scan. The diagnosis code G47.33 was assigned based on clinical judgment by physicians. Most patients did not undergo polysomnography (PSG), although a few cases had received PSG evaluation. Patients meeting either condition were included in the dataset (n = 5,480), whereas patients with missing BMI data were excluded (n = 607). Further exclusions included those with abnormal BMI values below 18.5 or above 60 (n = 147) and those under 50 years or over 85 years of age (n = 269). The final dataset consisted of 4,457 patients, which was used for preliminary and subgroup analyses (Fig 1).

### Detection of BMD and T-score

We used VeriOsteo™OP (Acer Medical Inc., New Taipei City, Taiwan), an AI-assisted screening software (version 1.00.3000) that utilizes deep learning to analyze the thoracolumbar region (T12-L1) in patients. We presented the results of key studies validating the performance of VeriOsteo™OP, which was approved by the Taiwan Food and Drug Administration and classified as a Class II medical device in Taiwan. The performance was validated by comparing the results with DXA assessments conducted in the medical centers. The operational principles and performance evaluation of VeriOsteo™OP were previously described [44].

### Independent variable and outcome variable

The variables used in this study included patient gender, age, BMI, BMD, and T-score (Bone Mineral Density Standard Deviation). To determine the association between osteoporosis severity and OSA, patient T-scores were categorized according to the recommendations of the International Osteoporosis Foundation (IOF) [46–48]. The categories were defined as “Normal” (T-score > −1.0), “Low Bone Mass” (−2.5 < T-score ≤−1.0), and “Osteoporosis” (T-score ≤−2.5) [8,25,38]. These variables were used to predict the likelihood of patients having OSA.

### Statistical analysis

Handling of quantitative and qualitative data: Age, BMI, BMD, and T-score were treated as quantitative data, whereas gender and T-score groups (0, if T-score > −1.0; 1, if −2.5 < T-score ≤−1.0; 2, if T-score ≤−2.5) were analyzed as qualitative data. Statistical analyses were used to determine the properties of the features, with separate tests conducted for qualitative and quantitative data.

Qualitative Data Analysis: The Chi-square test was used to assess the distributional differences of qualitative variables (e.g., gender and T-score groups) between patients with and without OSA. P-values were calculated to assess statistical significance.

Quantitative Data Analysis: For quantitative variables, such as age, BMI, BMD, and T-score, a t-test was used to compare the mean differences between patients with and without OSA. Mean values and standard deviations were reported.

Both statistical tests were two-tailed, with a significance level set at = 0.05. All statistical analyses were performed using Python version 3.11.11 with SciPy version 1.12.0.

### Machine learning

The dataset was divided into two subsets, a training set and a validation set, with a ratio of 9:1. The training set was used to train the models, and tenfold cross-validation was implemented to prevent the risk of overfitting. The validation set was used to assess model performance on the external data and to simulate whether the models would achieve comparable performance to that of the validation set. Several machine learning models, including logistic regression, random forest, and XGBoost, were used to predict the likelihood of patients having OSA [16,19,49–53]. The predictive performance of the models was evaluated using a Receiver Operating Characteristic (ROC) curve. The area under the curve (AUC) was calculated to compare the effectiveness of each model. In addition, by selecting optimal intervals for key variables, we identified ranges in which model performance could be further enhanced. The detailed protocol is available on Protocols.io at: https/dx.doi.org/10.17504/protocols.io.x54v9wbopl3e/v1.

## Results

Table 1 presents the qualitative statistical results of the study population. This study included a total of 4,457 patients, comprising 761 patients with OSA and 3,696 patients without OSA. The prevalence of OSA among male patients reached 45%, whereas that among female patients was approximately 8%. Regarding the T-score groups, the proportion of patients with OSA decreased as the severity of osteoporosis increased. The gender and T-score groups were significantly associated with the occurrence of OSA (p < 0.01).

**Table 1:** Qualitative Statistical Summary of the Patients.

		Overall (%)	With OSA	Without OSA	
		(n = 4,457)	(n = 761)	(n = 3,696)	p-value
Gender	male	1,064 (28.37)	484 (45.49)	580 (54.51)	<.0001
	female	3,393 (76.13)	277 (8.16)	3,116 (91.84)	
T-Score groups	0	659 (14.79)	262 (39.76)	397 (60.24)	<.0001
	1	2,320 (52.05)	424 (18.28)	1,896 (81.72)	
	2	1,478 (33.16)	75 (5.07)	1,403 (94.93)	

OSA: Obstructive Sleep Apnea.

A t-test was used to determine if significant quantitative differences existed between patients with and without OSA. Table 2 lists the quantitative statistical results for the study population, including age, BMI, and BMD, as well as engineered features, such as T-score. Patients with OSA exhibited a significantly higher BMI, BMD, and T-score compared with those without OSA. Conversely, the age of patients with OSA was significantly lower compared with that of patients without OSA. These findings suggest that these variables may hold potential value in predicting OSA risk.

**Table 2:** Quantitative Statistical Summary of the Patients.

		Overall (%)	With OSA	Without OSA	
		(n = 4,457)	(n = 761)	(n = 3,696)	p-value
Basic Information	Age	67.84 ± 9.38	62.47 ± 8.39	68.95 ± 9.20	<.0001
	BMI	26.01 ± 4.14	27.66 ± 4.52	25.67 ± 3.98	<.0001
	BMD	0.93 ± 0.11	1.01 ± 0.11	0.92 ± 0.11	<.0001
Feature Engineering	T-score	−2.02 ± 0.95	−1.37 ± 0.92	−2.16 ± 0.90	<.0001

μ represents the mean and σ represents the standard deviation. All values in the table are presented as μ ± σ for each group. BMD: Bone Mineral Density.

**Fig 2:**
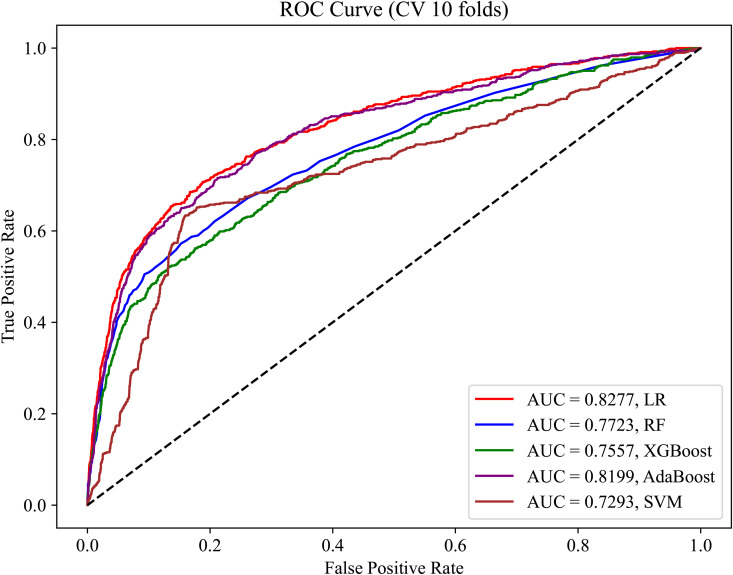
ROC curve of the training set without feature selection.

**Fig 3:**
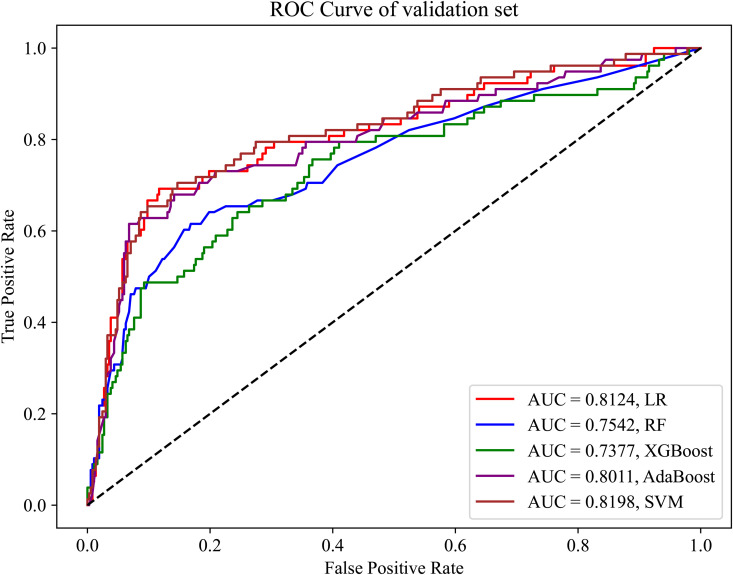
ROC curve of the validation set without feature selection.

Commonly used machine learning models were used to predict the likelihood of patients having OSA. Using the training dataset (variables: gender, age, BMI, and BMD), the predictive performance in terms of the AUC achieved approximately 75% or higher, with some models achieving up to 80% (Fig 2). To verify the generalizability of the models to external data, we evaluated the models using the validation dataset. As shown in Fig 3, most models exhibited a good performance on the validation dataset, confirming the stability of the models and their potential applicability to different scenarios.

**Fig 4:**
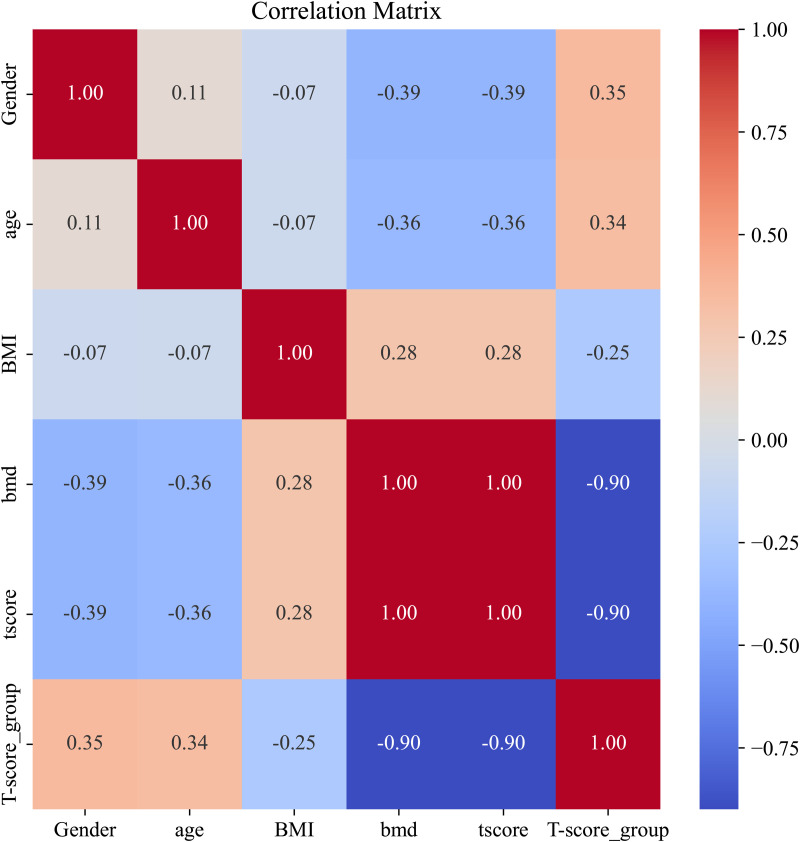
Correlation matrix without feature selection.

**Fig 5:**
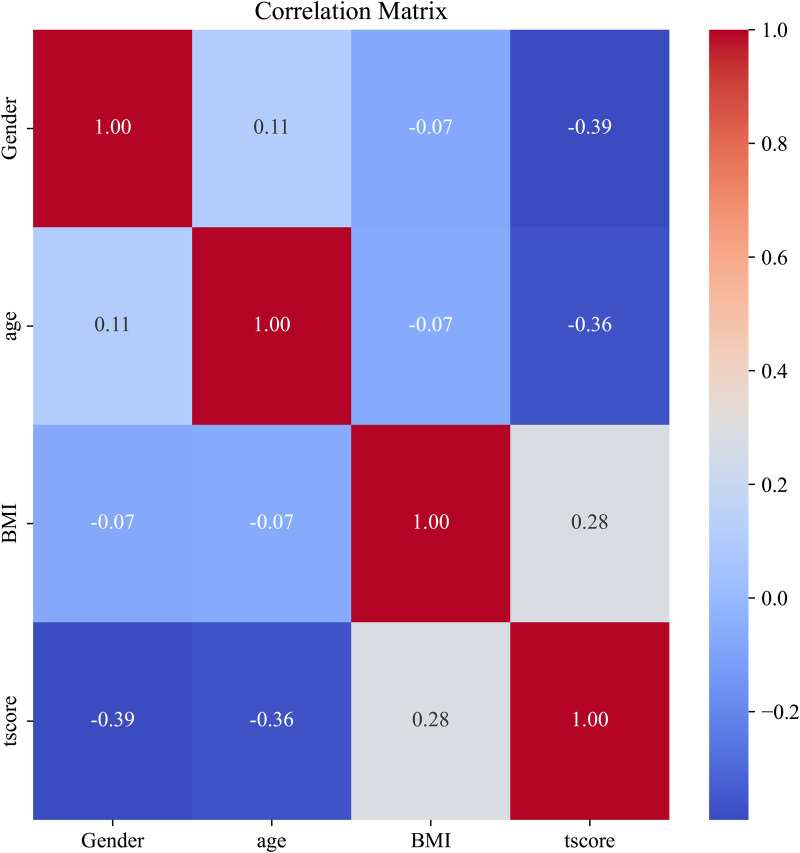
Correlation matrix with feature selection.

Before incorporating additional engineered features, we assessed the presence of collinearity between the variables. Because of the strong correlation among BMD, T-score, and T-score groups, a collinearity assessment was conducted using correlation matrices and the variance inflation factor (VIF). Following these analyses, significant collinearity between BMD, T-score, and T-score groups was evident (Fig 4; Table 3). Consequently, the BMD and T-score groups were removed as redundant variables. Postremoval, collinearity among the remaining variables fell within acceptable limits (Fig 5; Table 4).

**Table 3:** VIF Table Before Feature Selection.

Variable	VIF
Gender	1.1846
Age	1.1585
BMI	1.0945
BMD	1449.7682
T-score	1471.0134
T-score groups	5.2917

**Table 4:** VIF Table After Feature Selection.

Variable	VIF
Gender	1.1845
Age	1.1579
BMI	1.0927
T-score	1.4605

**Fig 6:**
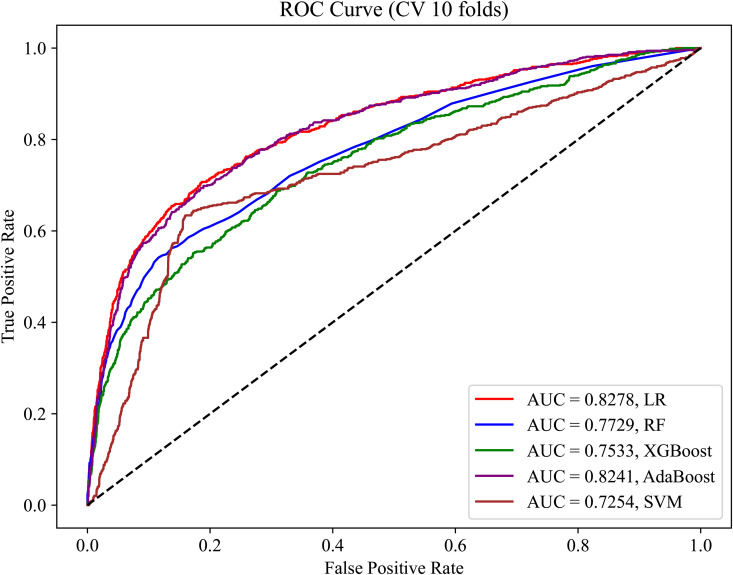
ROC curve of the training set with feature selection.

**Fig 7:**
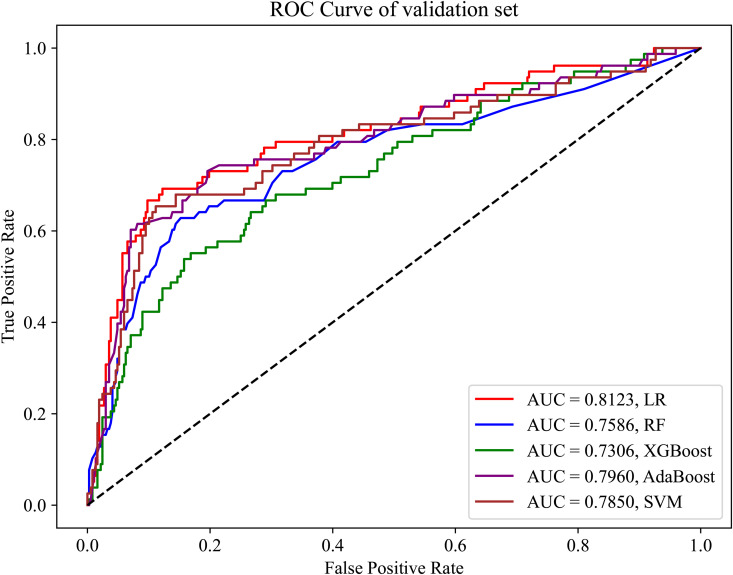
ROC curve of the validation set with feature selection.

Figs 6 and 7 show that after feature selection, the predictive performance of the models did not show significant improvement. Some models exhibited a decline in performance. This decrease may be attributed to the additional variables not being sufficiently informative for enhancing the predictive capability of the model.

We determined the optimal range for the quantitative variables, age, BMI, and T-score, in which the model could achieve a superior predictive performance. Based on the results presented in Tables 5–7, the selected age ranges of 50–60 years and 50–65 years demonstrated good model performance on the training dataset. However, when evaluated on the validation dataset, the performance of the 50–60-year range showed a significant decrease compared with the training set. Within this specific age group, model performance exhibited a noticeable degree of instability across different algorithms. Although the AUC for the Random Forest model decreased by only approximately 0.02—indicating relatively stable test performance—the SVM model showed a substantially larger drop of nearly 0.1, suggesting more severe overfitting. Overall, these findings highlight the potential challenges in model stability and generalization when conducting age-stratified analysis, and point to the need for further refinement in feature selection to improve consistency across age groups.

**Table 5:** AUC for the Selected Age Range.

Cross-validation of the training set					
	(50, 60)	(60, 70)	(70, 80)	(80, 85)	(50, 65)
LR	0.8334	0.7819	0.7364	0.7824	0.8254
RF	0.7877	0.7156	0.6837	0.7099	0.7796
XGBoost	0.7576	0.6896	0.6597	0.6747	0.7445
AdaBoost	0.8220	0.7779	0.7429	0.7615	0.8201
SVM	0.8004	0.7140	0.7035	0.7444	0.7787
Validation set					
	(50, 60)	(60, 70)	(70, 80)	(80, 85)	(50, 65)
LR	0.7622	0.7680	0.7797	0.4222	0.8228
RF	0.7697	0.7410	0.6782	0.2333	0.8057
XGBoost	0.7253	0.7166	0.6073	0.3556	0.7657
AdaBoost	0.7474	0.7628	0.7384	0.2000	0.8032
SVM	0.7080	0.7842	0.7992	0.2222	0.7762

LR: Logistic Regression, RF: Random Forest.

**Table 6:** AUC for the Selected BMI Range.

Cross-validation on the training set					
	(18.5, 24)	(24, 27)	(27, 30)	(30, 35)	(35, 60)
LR	0.7593	0.8411	0.8223	0.8266	0.8056
RF	0.6726	0.7954	0.7543	0.7754	0.7056
XGBoost	0.6542	0.7588	0.7502	0.7717	0.7357
AdaBoost	0.7606	0.8344	0.8068	0.8174	0.7756
SVM	0.7063	0.7899	0.7740	0.7800	0.6840
Validation set					
	(18.5, 24)	(24, 27)	(27, 30)	(30, 35)	(35, 60)
LR	0.6728	0.8608	0.8303	0.9111	0.7778
RF	0.6009	0.8350	0.7285	0.7944	0.7778
XGBoost	0.5595	0.8208	0.6398	0.7250	0.7778
AdaBoost	0.6520	0.8284	0.8107	0.9083	0.7778
SVM	0.6467	0.8316	0.7811	0.8806	0.7778

**Table 7:** AUC for the Selected T-score Range.

Cross-validation on the training set			
	(<−2.5)	(−2.5, −1)	(>−1)
LR	0.6322	0.7892	0.8003
RF	0.5764	0.7221	0.7431
XGBoost	0.5703	0.6906	0.7264
AdaBoost	0.6550	0.7846	0.7943
SVM	0.6054	0.7428	0.7630
Validation set			
	(<−2.5)	(−2.5, −1)	(>−1)
LR	0.6083	0.8081	0.8177
RF	0.4809	0.7454	0.7007
XGBoost	0.5408	0.6850	0.6931
AdaBoost	0.5735	0.7782	0.7805
SVM	0.5274	0.7794	0.8169

For BMI, the ranges of 24–27, 27–30, and 30–35 all exhibited satisfactory performance, with each achieving an AUC of 75% or higher for the training dataset; however, when examining the validation dataset, only the 24–27 BMI range maintained stable performance.

Regarding the T-score, selecting a range greater than −1 was considered; however, after applying this range, no significant improvement in model performance was observed in either the training or validation datasets. These findings suggest that while specific ranges for age and BMI can enhance model performance, T-score selection does not provide a notable benefit in this context.

We further examined the combined optimal ranges for the three quantitative variables, age, BMI, and T-score, to enhance the model’s predictive performance. The combination of BMI and T-score ranges resulted in a modest improvement in model performance on the training set, but demonstrated a significant improvement on the validation dataset.

For the other combinations, the results showed minimal changes in performance compared with the original dataset (Tables 8–10). These findings indicate that while T-score selection alone did not produce satisfactory results, incorporating T-score ranges with other variables, particularly BMI, significantly improved model effectiveness. This suggests that the interaction between BMI and T-score contributes to better model performance, and highlights the importance of considering combined variable selection strategies for enhancing predictive outcomes.

**Table 10:** AUC for the Selected BMI and T-score Ranges.

Cross-validation on the training set		Validation set	
	(24, 27) * (>−1)		(24, 27) * (>−1)
LR	0.8584	LR	0.9481
RF	0.7537	RF	0.8593
XGBoost	0.7438	XGBoost	0.8370
AdaBoost	0.8683	AdaBoost	0.8852
SVM	0.7841	SVM	0.8741

**Table 8:** AUC for the Selected Age and BMI Ranges.

Cross-validation on the training set		Validation set	
	(50, 65) * (24, 27)		(50, 65) * (24, 27)
LR	0.8301	LR	0.8018
RF	0.8054	RF	0.8037
XGBoost	0.7504	XGBoost	0.7645
AdaBoost	0.8295	AdaBoost	0.7806
SVM	0.7886	SVM	0.7954

**Table 9:** AUC for the Selected Age and T-score Ranges.

Cross-validation on the training set		Validation set	
	(50, 65) * (>−1)		(50, 65) * (>−1)
LR	0.7944	LR	0.8140
RF	0.7767	RF	0.7395
XGBoost	0.7439	XGBoost	0.6807
AdaBoost	0.7877	AdaBoost	0.7904
SVM	0.7772	SVM	0.8105

An additional phenomenon observed in Tables 5 − 10 is that, contrary to the common perception that random forest and XGBoost are better suited for predictive modeling, logistic regression, and support vector machine (SVM) were superior in this study. This finding is consistent with similar observations reported in other studies using analogous analytical models [54,55].

We hypothesize that this outcome may be the result of data imbalance, which can cause tree-based models to be biased toward the majority class. Another possible explanation is the limited number of variables in this study, leading to substantial overlap among samples in certain areas. This overlap may hinder tree-based models from effectively distinguishing between classes, thereby reducing their predictive accuracy.

## Discussion

Although the prevalence of obstructive sleep apnea (OSA) is higher among individuals over the age of 50, the observation that OSA patients tend to have a “younger” average age compared to controls may be attributed to the relatively low diagnosis rate. A key contributing factor is diagnostic delay. As people age and develop more complex health conditions, symptoms of OSA are often mistaken for other illnesses such as heart disease or hypertension, leading to misdiagnosis or underdiagnosis of OSA [56].

This study found that patients with OSA exhibited significantly higher BMD and T-scores compared to those without OSA, which seems to contradict the initial hypothesis that OSA may contribute to osteoporosis. This discrepancy may be due to the confounding effects of obesity, as obesity is not only a major risk factor for OSA but is also positively associated with higher bone density, potentially masking the adverse effects of OSA on bone health. In this context, the AI model may be predicting OSA based on the statistical association where patients with higher BMI tend to also have higher BMD and T-scores, rather than reflecting a direct physiological effect of OSA on bone integrity. Thus, while the model is capable of making predictions, the patterns it learns should be further validated by controlling for potential confounders to ensure robustness and generalizability.

In this study, we mitigated the influence of confounding variables by constraining specific ranges, aiming to identify whether the remaining variables are genuinely associated with OSA. Tables 5–7 illustrate the performance of models across different ranges for individual variables. The optimal age range for model performance was 50–65 years.

Regarding BMI, the most effective range was 24–27, which corresponds to mild obesity according to the standards of Taiwan’s Ministry of Health and Welfare [45]. The rationale for this optimal range may be that although OSA patients often exhibit obesity, severe obesity may result in discrepancies between the BMD predicted by VeriOsteo™OP and the actual BMD values. Because the T-score is derived from BMD, this discrepancy can lead to deviations in the model’s predictions of OSA.

Examining the intersection of two variable ranges (Table 10) revealed an interesting finding. When the range of the T-score is optimized alone, the performance gain is minimal; however, when combined with the BMI range, the model’s performance improves significantly. This outcome suggests that once the BMI effect is constrained, the information provided by the T-score becomes much more valuable. This indicates that there is indeed an association between the T-score and OSA, but this relationship is valid only under specific assumptions. For example, constraining the BMI range minimizes the interference of BMI on the model’s predictions, allowing the T-score to contribute more effectively to the identification of OSA.

In addition, the discussion highlighted that the predictive model developed in this study was particularly applicable within a specific body mass index (BMI) range, especially between 24 and 27, where the model showed its best performance. This observation is of notable significance. As shown in Table 11, both BMD and T-scores increase with higher BMI, regardless of whether patients had OSA or not. This indicates that BMI has a significant influence on bone health and may act as a confounding factor in the model’s predictions. In individuals with higher BMI, increased bone density could potentially mask the adverse effects of OSA on bone integrity, thereby complicating the model’s ability to accurately capture the true relationship between OSA and osteoporosis.

**Table 11:** BMD and T-score by BMI Groups in OSA and Non-OSA Patients.

Feature	BMI_group	Overall	With OSA	Without OSA
BMD	(18.5, 24)	0.89 ± 0.23	0.96 ± 0.25	0.89 ± 0.22
	(24, 27)	0.93 ± 0.21	1.01 ± 0.21	0.92 ± 0.20
	(27, 30)	0.96 ± 0.22	1.02 ± 0.21	0.94 ± 0.21
	(30, 35)	0.97 ± 0.22	1.04 ± 0.20	0.95 ± 0.20
	(35, 60)	0.99 ± 0.20	1.05 ± 0.19	0.96 ± 0.18
Feature	BMI_group	Overall	Without OSA	With OSA
T-score	(18.5, 24)	−2.34 ± 1.88	−1.79 ± 2.09	−2.4 ± 1.82
	(24, 27)	−2.01 ± 1.78	−1.39 ± 1.69	−2.13 ± 1.69
	(27, 30)	−1.8 ± 1.85	−1.27 ± 1.75	−1.95 ± 1.76
	(30, 35)	−1.68 ± 1.78	−1.15 ± 1.66	−1.86 ± 1.67
	(35, 60)	−1.5 ± 1.67	−1.04 ± 1.53	−1.79 ± 1.49

More importantly, referring back to Table 1, the average BMD and T-score values from the overall dataset (i.e., not stratified by BMI) are most closely aligned with those in the BMI 24–27 group from Table 11, for both the OSA and non-OSA groups. This suggests that the model may have learned the most stable and generalizable patterns from this BMI subgroup during training and validation, which could explain its superior performance in this range. This finding not only clarifies the model’s range of applicability but also underscores the importance of BMI as a potential confounder. Future studies should consider the influence of BMI stratification on model accuracy and assess its utility and generalizability across populations with varying body compositions.

### Strengths and limitations

The primary strength of this study lies in its use of CXR to predict BMD and combine this information with conventional clinical data to assess the likelihood of OSA. Compared with DXA, this approach does not require cumbersome procedures and the data are more readily available than those used in conventional OSA diagnostic methods. This technique holds significant potential for early OSA screening as it allows for the preliminary identification of high-risk populations without additional costs.

This study had several limitations. First, the VeriOsteo™OP software used to predict BMD is currently applicable only to individuals aged 50 years and older; however, as noted in the literature, the impact of OSA on BMD may not be as pronounced in older populations [36]. Second, data selection criteria included patients who had undergone DXA, which in practice, is more frequently performed on women. This gender imbalance in the dataset may not reflect the actual population distribution and could limit the generalizability of the findings. Larger datasets with more diverse demographics will enhance model performance for males and younger populations.

Third, the study utilized a limited set of variables, which may hinder the model’s ability to accurately delineate decision boundaries, potentially resulting in misclassifications. Fourth, the study population was restricted to patients from Tungs’ Taichung MetroHarbor Hospital, which may not be representative of the broader OSA patient population. Furthermore, the racial homogeneity of the study cohort may limit its applicability to other ethnic groups, thus constraining the generalizability of the findings.

Lastly, since the diagnosis of OSA in this study was based on ICD-10 codes, which are themselves reliant on physician judgment, the study is subject to potential diagnostic bias. Given this inherent limitation, OSA cases were identified solely through formally coded diagnoses. Future research could benefit from access to more comprehensive and granular datasets, which would enable more accurate identification and characterization of OSA.

### Future directions

In addition to its retrospective findings, the model proposed in this study demonstrates considerable potential as an early-warning, risk-predictive tool in clinical settings. It can be incorporated into routine patient evaluations to proactively identify individuals at elevated risk for obstructive sleep apnea (OSA), thereby enabling timely clinical intervention. By combining chest radiograph-derived bone mineral density (BMD) estimations with conventional clinical data, the model offers a non-invasive, accessible means for clinicians to assess the necessity of further diagnostic procedures—particularly polysomnography (PSG)—for selected patients. This predictive framework may improve the allocation of diagnostic resources by prioritizing patients more likely to benefit from PSG, thus enhancing screening efficiency.

Based on the aforementioned limitations, several key directions should be pursued in the future. First, the VeriOsteo™OP technology should be expanded to predict BMD in individuals aged 30–50 years. This would enhance the model’s predictive capability beyond specific age groups and allow for the early identification and treatment of at-risk populations, particularly younger individuals who may be at higher risk for OSA. Incorporating younger cohorts would improve the model’s robustness and applicability for early intervention.

Second, collaborations with additional healthcare systems are necessary to assess the model’s generalizability and performance across diverse clinical settings. Evaluating the model in various healthcare environments would ensure its adaptability and reliability, facilitating broader clinical implementation and improving its effectiveness for different patient populations.

## Conclusion

This study utilized CXR image analysis to predict BMD, which was subsequently used to investigate the association between osteoporosis and OSA, highlighting the potential application of AI technology in OSA-related risk screening. The integration of CXR imaging with machine learning models offers a radiation-free and cost-effective screening tool for clinical practice. Future studies should focus on further optimization of the algorithms to enhance predictive accuracy and reliability.
